# Lab-on-a-chip device made by autohesion-bonded polymers

**DOI:** 10.1007/s10544-017-0250-8

**Published:** 2017-12-18

**Authors:** Firas Awaja, Tsz-ting Wong, Benedicta Arhatari

**Affiliations:** 1 Department of Orthopaedic Surgery, Experimental Orthopaedics, Medical University Innsbruck, Innrain 36, Innsbruck, Austria; 2 Regenerative Medicine Institute (REMEDI) and CÚRAM Centre for Research in Medical, Galway, Ireland; 3 Department of Mechanical Engineering, The Hong Kong Polytechnic University, Hong Kong, Hong Kong; 4 Latrobe University, Bundoora, Melbourne, Australia

**Keywords:** Plasma treatment, Microfabrication, Sealing, Lab-on-a-chip, Microfluidics

## Abstract

Polymers have the obvious advantages of flexibility in design and cost effectiveness to fabricate a lab-on-a-chip (LOC) device. Polyether ether ketone (PEEK) in particular is very attractive choice as it adds biocompatibility in addition to the possibility of hematic sealing in a 3D design. Hereby, we extend our previous successful technology of autohesive hermetic bonding of medical implants into lab-on-a-chip devices. We explore a conceptual 3D micro channels design with hermetic potential using PEEK and PS sheets. A hermetic and mechanically strong (through tensile test) 3D multilayer device was obtained using plasma treatment with oxygen and methane as precursors followed by pressing at temperature near of T_g_ + 20 of the polymer with the lowest T_g_ (PS). This nanotexturing technique is also used to facilitate thermal and mechanical stability of the microchannels for microfluidic applications. X-ray tomography measurements showed that 3D polymer made chips, at certain plasma and press bonding conditions, have structural integrity and no deformation were detected in channels shape post thermal pressing process. The dimension stability of channels and reservoirs and the rigid interfacial region at PEEK-PS make this chip design attractive and feasible for advanced lab-on-a-chip applications.

## Introduction

Lab-on-a-chip (LOC), a group of micro total analysis systems (μTAS), is a microfluidic device integrated with micro or nano-sized multiplexed channels and circuits for biological sensing and diagnostic applications. It enables to perform fast, accurate and high-throughput analyses including cells and particles detection (Lenshof and Laurell 2010; Shields et al. 2015), nucleic acids sequencing (Nestorova et al. 2016; Kaprou et al. 2016; Mauk et al. n.d.), and protein separation (Li et al. 2014; Mesbah et al. 2016). DNA analysis, HIV and infectious disease testing are the fastest growing areas along with blood glucose monitoring achieving the largest point-of-care testing market share (Vashist et al. 2015).

Polymers offer cost-effective fabrication leading to high-quality and low-cost production over glass and silicon which have brittle nature and non-trivial sealing protocols making the fabrication process expensive and inaccessible. In addition, thermoplastics owning to the versatility are of special interest for the development of μTAS (Sackmann et al. 2014). New and advanced techniques can be exploited by taking advantages of the selective properties that the polymers possessed. Earlier methods to fabricate polymer microfluidic devices were the micromachining, hot embossing, injection moulding, and soft lithography. The microchannels constructed by these methods could reached the dimension at around 15-30 μm (Becker and Locascio 2002). Since then, multiple layers were required to be assembled with the use of different adhesion methods bonding the parts together permanently. Generally, the chip consisted of three parts which are a lid, a gasket, and a base (Hongbin et al. 2009; Melin et al. 2008; Weigl et al. 2003). This fabrication method was more economically effective than the equivalent microfluidic device produced using injection moulding. The multilayering or multiplexing strategy opens the possibility toward three-dimensional and multi-functional properties on the miniature devices (Lafleur et al. 2012).

Comparing the well-known capillary-driven platform, multiplexed microfluidic components within one single polymer material becomes inevitable to the pressure-driven microfluidic system (Haeberle and Zengerle 2007). Hermetic sealing of the LOC device for medical analysis is crucial in order to (1) confine the liquid samples for being tested, stored and transferred, (2) reduce the risk of contamination or biohazards, and (3) improve the accuracy of testing results. Polyether ether ketone (PEEK), a semi-crystalline thermoplastic, is being the favourable polymer following PDMS (polydimethylsiloxane) (Temiz et al. 2015). It can provide the LOC device with excellent chemical and hydrolysis resistance, mechanical and thermal stability (Diez-Pascual et al. 2012). The traditional polymer manufacturing techniques, such as injection moulding and soft lithograph, are not feasible to fabricate PEEK devices due to the high melting point whereas the conventional sealing methods, such as adhesives and high temperature, are not reliable for miniature devices which the residue causes clogging of microchannels and impedes the fluid flow (Arayanarakool et al. 2010; Bartolo et al. 2008; Dupont et al. 2010). Autohesion, a promising sealing technique, of thermoplastics can be achieved without the use of adhesives after suitable activation process with the applications of pressure and mild temperature.

Both aromatic PEEK and polystyrene (PS) thermoplastics obtain high mobility in a mould between the glass transition temperature (T_g_) and meting temperature (T_m_) and become rigid in a shape upon subsequent cooling below T_g_ retaining the dimensional, chemical and mechanical properties over a range of operational temperatures and pressures. Optical transparency of LOC device is desirable for the purpose of visual inspection, in this point of view, PS is the preferred thermoplastic especially when bonds particularly after oxidizing treatment with PEEK polymer to provide a good structural integrity (Lu and Weiss 1996). Autohesion of PS was firstly introduced by Shtarkman et al. in 1965 (Shtarkman et al. 1965). Cho et al. in 1995 published the findings on the effects of processing parameters to the bonding strength of PEEK (Cho and Kardos 1995). Both studies demonstrated the autohesion of the polymers were satisfactorily achieved at relatively high temperatures above T_g_. Autohesion of PEEK at lower temperature near T_g_ can be achieved using plasma treatment with the help of oxidizing gases. Processing temperature below T_g_ is favourable as to ensure no polymer deformation of the microchannels during sealing process. Moreover, autohesion achievement is highly dependent on the gas composition and plasma condition. According to the previous studies, the autohesive bonding strength of PEEK can reach a double increased through a single oxygen or methane plasma treatment while the PEEK can even reach a higher autohesive bonding strength through the mixture of methane/oxygen plasma treatment (Arias-Zapata et al. 2016; Zhang et al. 2011). In addition, oxygen plasma has been used for the pretreatment method to remove the contaminants and forming the polar functional groups on the polycarbonate surface and therefore improve the adhesion of deposition coating (Guo and Hong 2002).

The aim of this study is to demonstrate a time-and-cost-effective production of LOC devices through the autohesion of PEEK and PS using oxygen and methane plasma treatments at a temperature near T_g_. A LOC device with hermetic seal and structural integrity is achieved without the use of adhesives, delamination at interfaces and clogging of microchannels for microfluidic applications.

## Experimental

### Materials

**Fig. 1:**
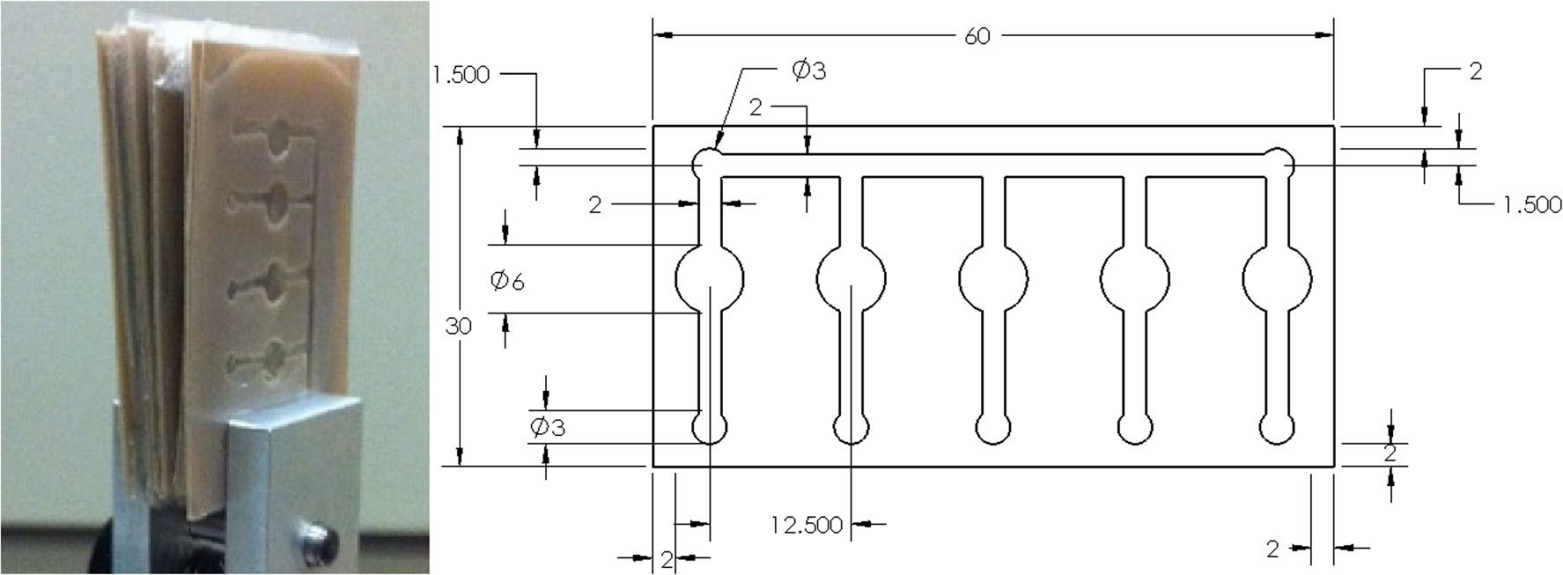
The PEEK-PS lab-on-a-chip devices (left) and the technical design drawing of the chip including the micro-channels and the reservoirs (right)

A semi-crystalline polyether ether ketone (PEEK) film with the thickness of 500 μm was sourced from Victrex plc, UK. The PEEK film was cut into rectangular samples with the dimension of 60 mm × 30 mm and they were then cut into a design with channels and reservoirs as shown in Fig. 1. An aromatic polystyrene (PS) sheet with the thickness 250 μm was cut into rectangular samples with the same dimension of 60 mm × 30 mm. All samples were washed using 100% ethanol, dried under room temperature and then wrapped using aluminum foil and stored in desiccator to prevent the samples from contamination. The glass transition temperature (T_g_) of PEEK and PS were evaluated using Temperature Modulated Differential Scanning Calorimetry (TMDSC) with the measurement parameters of heating ramp and modulation period set according to the recommended specifications (Thomas 2005). The results showed PEEK and PS have their T_g_ at 150 °C and 95 °C respectively.

### Plasma treatment on PEEK and PS

Plasma immersion ion implantation (PIII) technique was applied on PEEK and PS surfaces using the activation system set up introduced previously (Awaja et al. 2010a, b). The system consisted of a radiofrequency (RF) power supply of 13.56 MHz coupled via an impedance matching network with an externally mounted antenna located in the plasma chamber attached to the treatment chamber. The sample stage, same as the treatment chamber, using stainless steel was mounted on a glass tube in order to produce an electrical isolation to induce potential switching between biased or left at floating potential to the sample.

The plasma reactor system applied the base pressure of 7 × 10^−3^ Pa. The RF power supply was operated between 100 and 150 W with the reflected power ranging from 25 to 50 W. The sample stage was connected to the biased voltage functioning between 2 and 10 kV at the frequency of 2000 Hz. Oxygen and methane plasma gases of highly purity were introduced into the treatment chamber with the same flow rate of 38.6cm^3^/min under the operating pressure of 0.64 ± 0.02 Pa.

PEEK and PS samples were initially cleaned with ethanol and placed on the sample stage. The treatment chamber was evacuated to based pressure and cleaned with oxygen plasma gas for 5mins before the plasma treatment. The plasma treatment on samples was considered as effective after 30s. The samples were then treated with methane plasma gas for 4mins at 100 W and 150 W power with 50 W reflected. After the plasma treatment, the chamber was depressurized filling with atmospheric air. The plasma treated samples were wrapped using aluminum foil and stored in desiccator for 20 h prior to autohesion process in order to ensure the uniform aging performed on the plasma treated surfaces.

### Autohesion process

The chip configurated with a PEEK separator gasket and two PS outer layers was pressed together using a temperature controlled hot press machine, Tetrahedron MTP-14 (Tetrahedron Associates, Inc., USA), under the pressure of 653 kPa for 2 h. Eight PEEK-PS chips were bonded at different temperatures of 75 °C, 85 °C, 95 °C, 105 °C, 115 °C, 125 °C, 130 °C and 165 °C at the heating rate of 5 °C/min. Five PEEK-PS chips bonded at each temperature were prepared for further ethanol leak out and x-ray tomography tests.

### Ethanol leak out test

A hole was created at the bottom of each channel using a 29-gauge insulin syringe with needle of 0.33 mm diameter. 0.2 ml ethanol was injected into the middle channel, where produces the least resistance and stress onto the chip, through the needle to test the sealing condition of the chip. Afterward, the needle hole was sealed using Selleys repair glue. Each sample was weighed at regular time to monitor the mass loss.

### X-ray tomography test

X-ray tomography test was conducted using X-ray Micro Computed Tomography instrument (Xradia Inc., USA) from the Department of Physics at La Trobe University. X-ray micro computed tomography (XμCT), a non-destructive 3D imaging technique, is used to inspect the inner structure of an object using X-ray for transmission and measurement. The x-ray source with closed tube and tungsten target was operated at 60 kV tube voltage and 8 W power for the characterization of seven PEEK-PS chips at different bonding temperatures. The measurement parameters together with that set for the alternative measurement of individual samples at bonding temperature of 115 and 125 °C were shown in Table 1. The way the samples mounted in the micro-tomography instrument applies compression force from one end that lead to the chips to have a slight tilt. However, the samples are flat and they were manufactured from flat sheets of polymers.

**Table 1:** The measurement parameters of different PEEK-PS chips

	Low resolution		High resolution
	Eight PEEK-PS chips at different bonding temperatures of 75 °C, 85 °C, 95 °C,105 °C, 115 °C, 125 °C, 130 °C and 165 °C	PEEK-PS chips at bonding temperature of 115 °C and 125 °C	PEEK-PS chips at bonding temperatures of 115 °C
X-ray energy	60 kV, 8 W	40 kV, 8 W	80 kV, 10 W
Exposure time for each projection	10s	10s	60s
Total number of projections	361	361	721
Objective magnification	0.5×	0.5×	10×
Source to sample distance	100 mm	120 mm	100 mm
Detector to sample distance	50 mm	20 mm	25 mm
Voxel number	948x1024x1024	948x1024x1024	1012x1024x1024
Effective voxel size	36 μm	46.3 μm	2.1 μm

CCD camera (Andor Technology Ltd., UK) coupled with a scintillator system and 0.5× objective de-magnified lens was equipped as the imaging detector. The camera has 2048x2048pixels with 13.5 μm physical pixel size. 2× binning was used in the data acquisition to reduce the size of the collected data set. The effective pixel size for the current setup was 36 μm on account of the geometric magnification and the distance of source-sample-detector. The Xradia software, TXMController, was used to control the setup of the tomography machine and data acquisition. Numbers of projection images were obtained by rotating the sample in order to collect the 3D data set. Each sample was scanned to acquire 361 projection images at equal angle interval in an angular range of 180°. Each projection image was recorded in 10s and corrected to dark current image for non-uniform illumination in the imaging system. A filtered back projection algorithm installed in the software of TXMReconstructor was used to reconstruct the acquired data for visualizing into 3D image. The total reconstructed volume contained 948x1024x1024voxels with 36 μm^3^ voxel size. After the data reconstruction, linear attenuation coefficient and 3D map were obtained within the sample section exposed by radiation. TXM3DViewer software was used to view the completed 3D data which the 3D map was proportional to the object density. Avizo-6.2 software from Mercury Computer System was used to automatically segment the 3D data based on gray scale to distinguish between empty space (reservoir and channel) and filled space (the chip) for further sample analysis. Each segmented region was individually assigned with different colour and the volume of each segmented region was calculated by the software.

While autoclaving which is one of the effective methods for processing sterilization on medical devices, chlorine dioxide gas (Girouard Jr and Czarneski 2016), gamma ray (International Atomic Energy Agency 2008) and electron beam sterilization (Medical Plastics and Biomaterials Magazine 1997) can be the effective alternatives for medical devices containing semiconductors that cannot sustain the high temperature and moisture necessary for autoclave/steam sterilization. These methods can be processed at low temperature and compatible with electronics.

## Results and discussion

The main objective of this work is to utilize our previous technology of bonding polymers near or under the glass transition temperature meant for medical implants to the lab-on-a-chip devices. We aimed at developing the autohesion bonding to enable a multilayer 3D design containing hermetic channels of the chip. We anticipated that bonding temperature as well as the plasma treatment conditions used for autohesion are the important factors to be considered. The integrity and shape of the channels were determined by X-Ray tomography.

### Bonding temperature effects

The chip was configurated with a polyether ether ketone (PEEK) middle core and two polystyrene (PS) outer layers. The interfacial surfaces of PEEK and PS were modified by methane PIII plasma treatment and bonded through autohesion at different bonding temperatures under the same heating rate and pressure. The results of PEEK-PS chips from microfluidic channel fabrication process were shown in Table 2 **.**

**Table 2:** The results of PEEK-PS chips from microfluidic channel fabrication process at different bonding temperatures

Bonding temperature	Channel impedance	Pierce ability using needle	Observation on the PEEK-PS chips after hot press
75 °C	No	Yes	No bonding Separated once opening the hot press machine
85 °C	No	Yes	Weak bonding Separated upon handling the chip
95 °C	No	Yes	Slightly stronger bonding than the samples at 85 °C bonding temperature
105 °C (sample E)	No	Yes	Structurally stronger bonding than the samples at 95 °C bonding temperature
115 °C (sample F)	No	Yes	The strongest bonding among the samples
125 °C (sample G)	Yes	No	PS layers became brittle after hot press The injection of ethanol would separate PS layers from PEEK under certain injection pressure
130 °C	Yes	No	Channel impedance occurred Greater flow resistance against ethanol than the sample at 125 °C bonding temperature
165 °C	Yes	No	PS layers became very brittle The highest channel impedance among the samples The greatest flow resistance against ethanol among the samples

The adhesion strength is determined by the interfacial bonding strength of PEEK-PS, nevertheless, the tensile strength of the PS along the channels and reservoirs is determined by the molecular strength of PS. Therefore, the bonding temperature at T_g_ + 20 °C of PS which is 115 °C was observed to obtain the strongest adhesion strength of PEEK-PS and tensile strength of PS amongst the samples such that the channels and reservoirs allow to function as a LOC device. Furthermore, amongst the samples, only PEEK-PS pressed at 115 °C showed no leak or permeation through null mass lose (after 24 h) measured by the ethanol leak out test. Suggesting a hermetic seal of the chip. Samples bonded at 115 °C showed no adhesive failure in a tensile strength test and only cohesive frailer were observed when the forces used exceeding 5 MPa.

The chips that were pressed below the T_g_ of PS were found to exhibit weak bonding behaviour. Further, no bonding was found between PEEK and PS at the bonding temperature below 85 °C. An intact chip could be obtained from the press machine at the bonding temperature of 95 °C but the adhesion failure happened upon handling. The chips pressed at and above 105 °C received an improvement in the bonding strength such that the ethanol leak out test could be conducted on the chips. It was discovered that, during the injection of ethanol into the chips, the needle separated the PS from PEEK at the injecting region and then it spread over the entire chip. There was a failure occurred for the PS layer due to the thermal deformation at higher temperatures. As there is a large area of free-standing PS layers along the channels and reservoirs inside the chip, the collapse of PS layer that clogged the channels has to be prevented particularly in the reservoirs. The chips pressed at and above 125 °C exhibited a significant collapse clogging both the channels and reservoirs which made the injection of ethanol into any parts of the chip impossible and the leak out test was unable to be conducted.

Contrarily, the increase in mass of some chips were identified during the ethanol leak out test. This could be explained by the water absorption of the repair glue used for sealing the needle hole during the curing process since the moisture air was allowed to interact with the chips due to the opening of desiccator.

### Integrity determination criteria

**Fig. 2:**
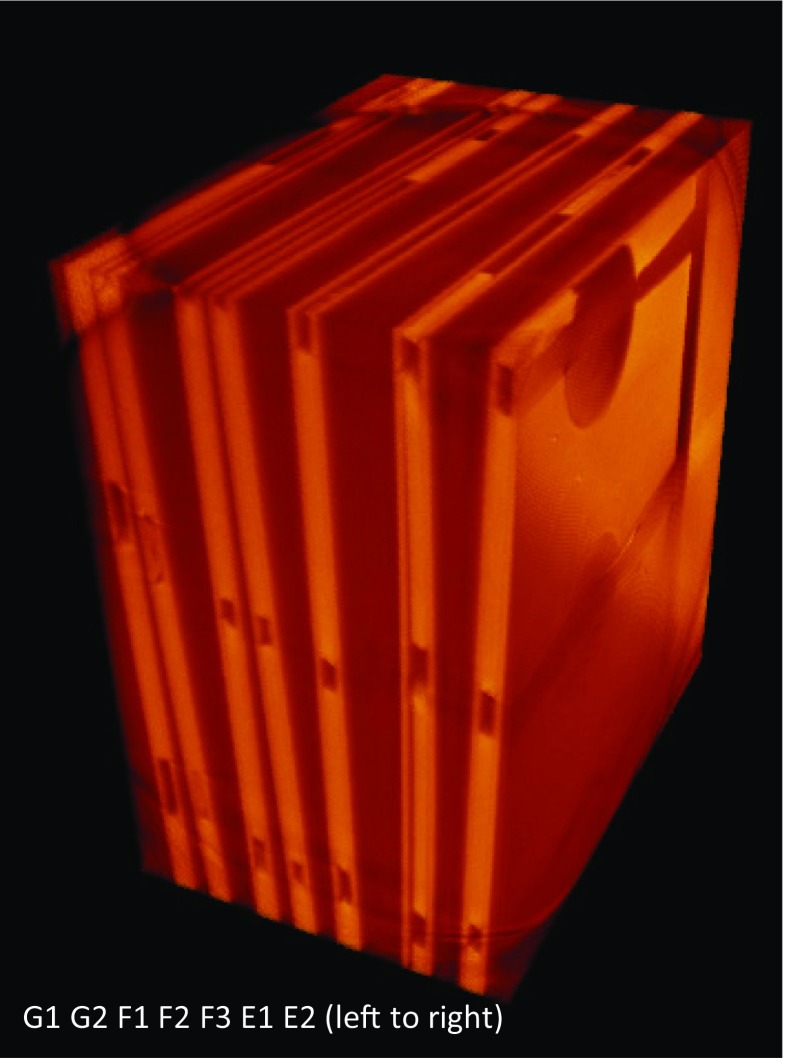
3D view of the chips bonded at 105 °C (E1, E2), 115 °C (F1, F2, F3), and 125 °C (G1, G2), respectively

**Fig. 3:**
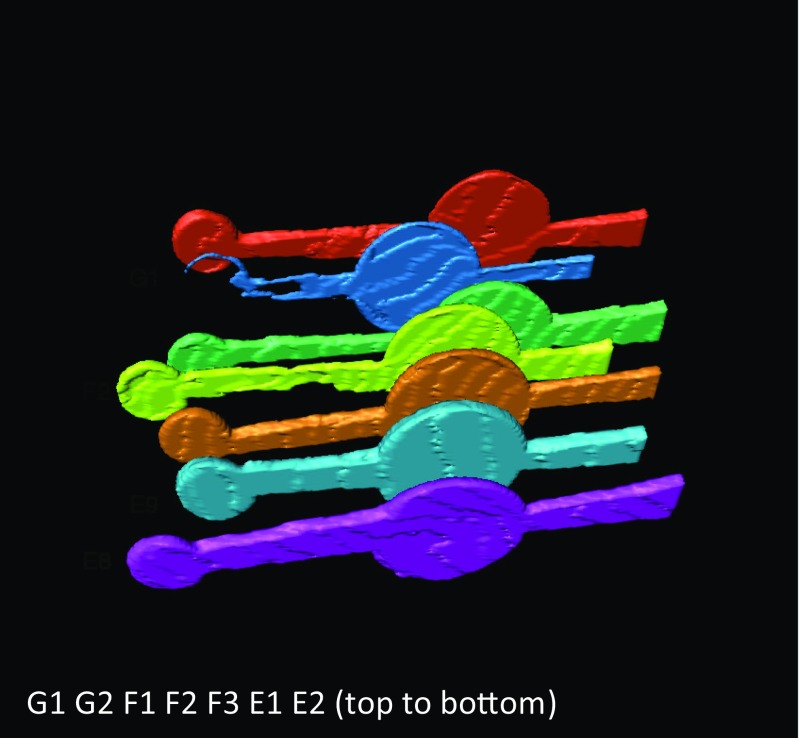
3D rendering of the volume inside the chips bonded at 105 °C (E1, E2), 115 °C (F1, F2, F3), and 125 °C (G1, G2), respectively

The integrity of the chip design was determined by several criteria which are the dimension stability of channels and reservoirs, interfacial condition at PEEK-PS, and interdiffusion performance at interfaces. The chips denoted as E1, E2, F1, F2, F3, G1, and G2 presented in Fig. 2 were the samples bonded at 105 °C, 115 °C, and 125 °C. They were analyzed through 3D imaging and rendering using XμCT techniques. The three-dimensional rendering shown in Fig. 3 demonstrated the volume in the chips (G1 and G2) bonded at 125 °C were particularly intact, in comparison with other samples. Samples processed at Temperature of 115 °C showed minimal deformation in the channels shape.

**Fig. 4:**
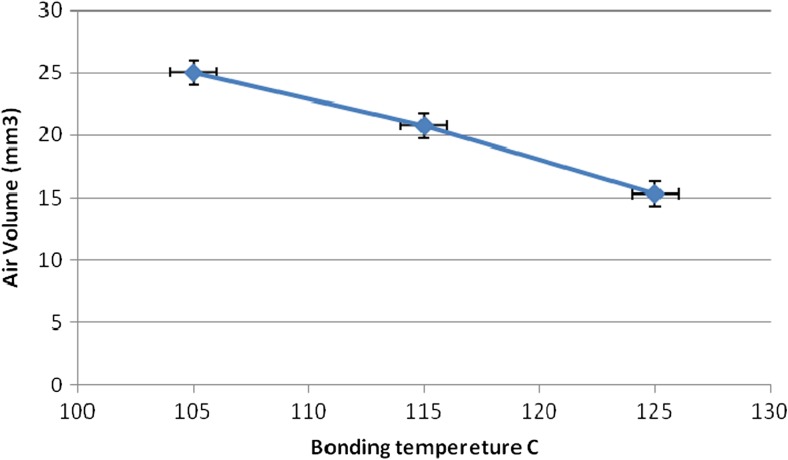
Air Volume inside the chips bonded at 105 °C, 115 °C and 125 °C

Figure 4 shows quantitative information that presented the decrease of air volume with the increase of bonding temperature of the chips. Every 5 °C the bonding temperature increased, about 5mm^3^ the air volume inside the chip decreased. The air volume inside the chip is relative to the dimension stability of the channels and reservoirs. The results indicated the thermal deformation of free-standing PS layers along the channels and reservoirs and the undesirable dimension stability of the chips bonded at 125 °C.

**Fig. 5:**
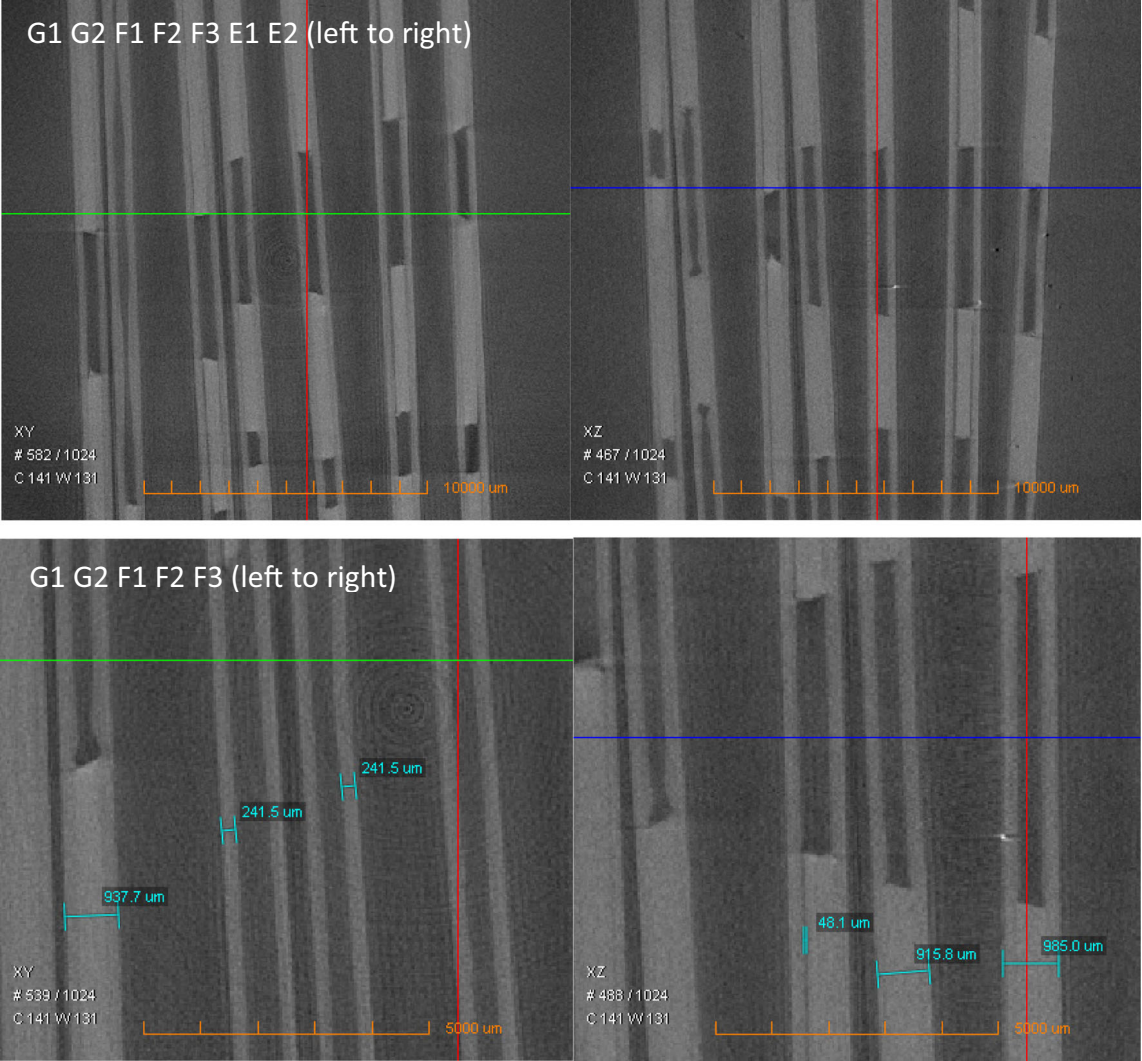
The dimension stability of the chips bonded at 105 °C (E1, E2), 115 °C (F1, F2, F3), and 125 °C (G1, G2) observed using tomography

Delamination was found to severely happen on the chips (E1 and E2) bonded at 105 °C according to the tomography images shown in Fig. 5. The thicknesses of the chip, PS layers, and delamination gap were measured. The chips and PS layers were measured to have a general thickness of 950 μm and 241.5 μm respectively. One out of the three chips was found delaminated with 48.1 μm wide at the bonding temperature of 115 °C, nevertheless, in overall, the channels and reservoirs inside the chips maintained the integrity and the adhesive behaviour of PEEK-PS performed as ideal.

**Fig. 6:**
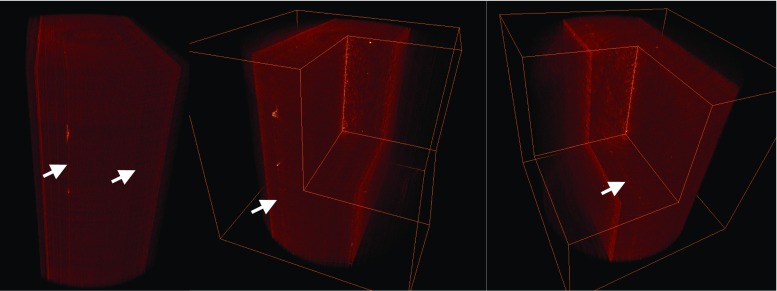
3D view indicating the interfaces (arrows) at PEEK-PS of the chip (F2) bonded at 115 °C observed under XμCT

**Fig. 7:**
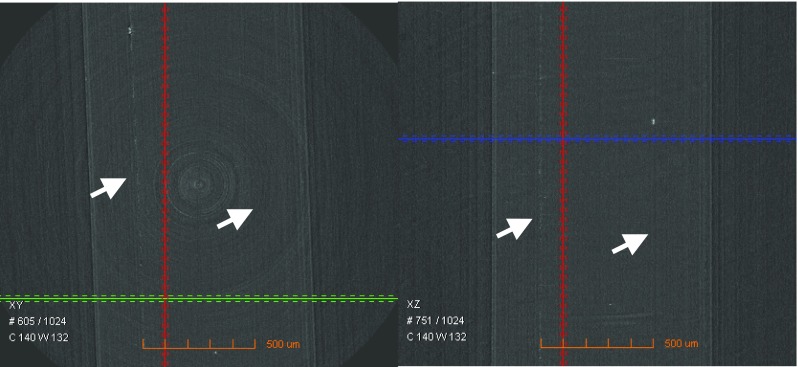
The interfaces (arrows) at PEEK-PS of the chip (F2) bonded at 115 °C observed under tomography

The chip (F2) bonded at 115 °C was further examined on the interdiffusion behaviour at the interface of PEEK-PS using XμCT technique. The images were shown in Figs. 6 and 7. Since the density of PEEK and PS are similar leading it difficulty in identifying the interface of PEEK and PS were perfectly bonded. The interdiffusion of the chip was evident and that the two polymers were entirely bound over their interfaces. Amongst all the samples, the chip bonded at 115 °C demonstrated excellent adhesion performance according to the XμCT images and maintained the integrity with excellent dimension stability of the channels and reservoirs for the functions of a LOC microfluidic device.

The proposed PEEK-PS microfluidic system demonstrated a highly effective production process through the plasma treatment on targeted surface and compression for self-adhesion. Unlike PDMS-PS (Arias-Zapata et al. 2016; Lee et al. 2016), no solvent chemicals are required. A solvent-free self-assembled dimensionally-stable PEEK-PS microfluidic system is possible.

## Conclusion

Microchannels integrity of the lab-on-a-chip (LOC) configured with a polyether ether ketone (PEEK) middle core and two polystyrene (PS) outer layers was successfully implemented through the autohesion of three polymer sections by using oxygen and methane plasma treatments. The optimal processing temperature was found at a low temperature near T_g_ of 115 °C. Higher bonding temperature caused polymer deformation and structure collapse clogging the microchannels. Conversely, lower processing temperatures lead to poor autohesion of polymers resulting into unstable LOC device. X-ray micro computed tomography (XμCT) with more powerful analyzing performance showed that most samples had intact channels and reservoirs, except for the samples processed at 105 °C. Based on the present findings from ethanol leak out test, XμCT characterization, no ethanol leakage and structural deformation were resulted on the PEEK-PS processed at 115 °C. These suggest a LOC device with hermetic seal and structural integrity is developed at a temperature near T_g_ without the use of adhesives, delamination at interfaces and clogging of microchannels for microfluidic applications.

Evidently, plasma treatment for PEEK and PS to achieve adequate hermetic and strong adhesion require sophisticated plasma device. Bonding temperatures for such manufacturing technique is strongly dependent on the glass transition and the melting temperatures of the selected polymers. These could be considered as limitation to the described technique. However, the gain advantageous of producing chips with clean and precise seal without the need for toxic adhesives make this technology attractive for applications in which microfluidic channels must be completely, cleanly and tightly encapsulated.
